# Children with facial paralysis due to Moebius syndrome exhibit reduced autonomic modulation during emotion processing

**DOI:** 10.1186/s11689-019-9272-2

**Published:** 2019-07-10

**Authors:** Elisa De Stefani, Martina Ardizzi, Ylenia Nicolini, Mauro Belluardo, Anna Barbot, Chiara Bertolini, Gioacchino Garofalo, Bernardo Bianchi, Gino Coudé, Lynne Murray, Pier Francesco Ferrari

**Affiliations:** 1 Unit of Neuroscience, Department of Medicine and Surgery, University of Parma, Via Volturno, 39, 43125 Parma, Italy; 2 Unit of Audiology and Pediatric Otorhinolaryngology, University Hospital of Parma, Parma, Italy; 3 Department of Humanities, Social Sciences and Cultural Industries, University of Parma, Parma, Italy; 4 Maxillo-Facial Surgery Division, Head and Neck Department, University Hospital of Parma, Parma, Italy; 5 Institut des Sciences Cognitives Marc Jeannerod, CNRS, Université de Lyon, Bron, France; 6 Department of Psychology, University of Reading, Reading, RG6 6AL UK

**Keywords:** Moebius children, Facial expressions, Autonomic nervous system, Thermal infrared imaging, Respiratory sinus arrhythmia, Emotion recognition

## Abstract

**Background:**

Facial mimicry is crucial in the recognition of others’ emotional state. Thus, the observation of others’ facial expressions activates the same neural representation of that affective state in the observer, along with related autonomic and somatic responses. What happens, therefore, when someone cannot mimic others’ facial expressions?

**Methods:**

We investigated whether psychophysiological emotional responses to others’ facial expressions were impaired in 13 children (9 years) with Moebius syndrome (MBS), an extremely rare neurological disorder (1/250,000 live births) characterized by congenital facial paralysis. We inspected autonomic responses and vagal regulation through facial cutaneous thermal variations and by the computation of respiratory sinus arrhythmia (RSA). These parameters provide measures of emotional arousal and show the autonomic adaptation to others’ social cues. Physiological responses in children with MBS were recorded during dynamic facial expression observation and were compared to those of a control group (16 non-affected children, 9 years).

**Results:**

There were significant group effects on thermal patterns and RSA, with lower values in children with MBS. We also observed a mild deficit in emotion recognition in these patients.

**Conclusion:**

Results support “embodied” theory, whereby the congenital inability to produce facial expressions induces alterations in the processing of facial expression of emotions. Such alterations may constitute a risk for emotion dysregulation.

## Background

When individuals are exposed to emotional faces, they spontaneously react with distinct electromyography responses in emotion-relevant facial muscles, a mechanism termed “facial mimicry” [1–4]. Notably, artificially interfering with participants’ spontaneous facial muscular activation during observation of facial expressions significantly reduces emotion recognition performance [5–7]. This evidence indicates a close relationship between the ability to express facial emotions and the ability to recognize facial expressions displayed by others [5, 8]. According to motor theories of perception, the observation of others’ facial expression activates the sensorimotor representations involved in the execution of that expression, facilitating recognition processes [9]. In particular, information concerning one’s own emotion is hypothesized to be retrieved through both somatovisceral and motor re-experiencing of an observed emotion [10].

The “mirror neuron system” (MNS) is considered part of the neurobiological substrate supporting this shared representation [11–13]. When we observe an individual performing an action, our motor cortices become active in the same way as if we were experiencing that action ourselves [14]. This simulation mechanism is useful for understanding others’ actions and goals within a motor framework [15–18] and can be applied to the domain of language and emotional development [19–23]. Although neuroimaging investigations have shown that a number of cortical and subcortical areas (involving the premotor cortex, the anterior cingulate cortex, and the anterior insula) that support first-person experience of a specific emotion also become active during the observation of that emotion in others [24–29], the debate concerning the role of simulation processes in emotional recognition remains an open one in the literature.

The study of facial expression processing in patients with peripheral facial palsy could be a potentially powerful empirical strategy for assessing simulation processes in emotion recognition. Among facial palsies, Moebius syndrome (MBS) is the most interesting condition, because it is present from birth and is characterized by weakening or paralysis of the facial muscles. The cranial nerves that are predominantly involved in this extremely rare syndrome (1/250,000 live births [30]) are the sixth and seventh; these directly control lateral eye movements and facial muscles, respectively [30]. These nerves are either absent or underdeveloped, resulting in bilateral or unilateral facial palsy. MBS is sometimes associated with musculoskeletal abnormalities and other cranial nerve palsies: these include, most commonly, the hypoglossal nerve [31], which often leads to atrophy of the tongue and, accordingly, speech problems [31]. Some patients with MBS may also present with additional deformities, such as orofacial, limb, and musculoskeletal malformations, whereas the patient’s intelligence is usually preserved [29, 31–35].

The diagnosis of MBS is based exclusively on clinical criteria. The classical diagnostic criteria are bilateral facial paralysis affecting both sides of the face (seventh cranial nerve) and paralysis of sideways (lateral) movement of the eyes (sixth cranial nerve) [29–32]. Recently, cases with unilateral facial palsy have also been included in the spectrum of this disease [30, 31, 33]. Because of their congenital lack of capacity for facial mimicry, the study of children with MBS is of great relevance for investigating the contribution to emotion recognition of facial simulation processes.

Here, we focus on an under-investigated topic: the contribution of the capacity for facial mimicry to autonomic regulation in response to others’ emotions. In fact, the autonomic nervous system (ANS) regulates the physiological reactions of the entire body to environmental stimuli [36], fostering either prosocial (e.g., a parasympathetic calm and relaxed state [37, 38]), or defensive (e.g., sympathetic fight or flight responses) behavioral strategies. The centrality of the ANS in emotion has been demonstrated in a large body of research aimed at assessing different aspects of the ANS–emotion relationship (for a review, see [39]), and the link between motor simulation and ANS reactivity is supported by several neuroimaging studies demonstrating how observation of others’ emotional facial expressions activates not only motor pathways [28], but also brain structures (e.g., amygdala, insula) [24, 25, 40] regarded as part of the extended MNS [41, 42], and thought to be responsible for emotional information processing. Despite the volume of studies, investigations of the effects of deficits in facial mimicry on autonomic regulation are still lacking. We hypothesized that MBS patients might present an alteration in autonomic responses to emotional stimuli as a consequence of the inability to express emotions from birth.

Among several techniques commonly adopted for ANS recording, functional infrared thermal imaging (fITI) and electrocardiography (ECG) were implemented in the present study. fITI is a technology that offers the advantage of a non-contact approach, which is suitable for human psychological and physiological studies [43]. fITI records the body’s naturally emitted thermal irradiation, which depends on cutaneous blood perfusion controlled by the ANS innervating the vessels that irrigate the skin [43]. Recently, it has been demonstrated that many emotional states are associated with variations in facial temperature [44–46]. Specifically, measuring thermal effects of emotional arousal may provide useful information about the sympathetic branch of the ANS, since skin temperature depends on cutaneous blood perfusion and local tissue metabolism, and sudomotor responses, all of which are controlled by the sympathetic system.

Using ECG, we estimated respiratory sinus arrhythmia (RSA) reactivity. RSA is a metric of heart rate variability associated with spontaneous breathing. RSA measures the parasympathetic branch of the ANS via cholinergic vagus nerve projections to the heart. During situations where active coping, or emotional regulation, is required, the vagal input increases RSA, supporting a flexible coping response. According to the polyvagal theory, this response is a physiological indicator of the individual’s ability to engage in appropriate regulatory behavior and provides a physiological substrate for affect regulation, which presumably underlies adaptive interpersonal functioning [47, 48]. Specifically, the vagal tone at rest is considered a stable neurophysiological mechanism reflecting potential autonomic reactivity in the absence of environmental challenge. In the literature, high resting RSA has been associated with appropriate emotional reactivity and indexes of the functional ability to engage and disengage with the environment [49].

We conducted two experiments testing for emotional processing in children with MBS. In the first experiment, we tested whether, compared to a non-affected control group of the same age, children with MBS were able to recognize stimuli representing facial expressions. In fact, in the literature, there are no studies on facial recognition of emotions in children with MBS, but only in adult patients, and results are inconclusive [50–52]. We used dynamic stimuli that, in the literature, have proved more effective than static images in inducing an emotional response [53–55]. The stimuli were facial expressions representing emotions of disgust, surprise, anger, and happiness. These emotions were selected based on the developmental stage of the participants. Thus, although even newborns are able to produce facial expressions [56], the ability to recognize specific emotions from facial expressions increases with age [57, 58]. Previous studies reported that, among the basic facial expressions, the emotions that are best recognized (from an actor’s full face display) are happiness, anger, and disgust, followed by fear, with sadness being more difficult to recognize [59–61]. More specifically, research has shown that, by 5 years of age, children are as sensitive as adults to displays of happiness, [62], and from 8 to 11 years, they recognize happy, angry, and disgust expressions more easily than those showing fear and sadness [62, 63]. For these reasons, among the basic facial expressions, we included two positive emotions (happiness and surprise) and two negative emotions (anger and disgust), whereas facial expressions of fear and sadness were discarded.

Once the ability of children with MBS to recognize facial expressions was ascertained, we determined whether the emotional processing and the responses of the ANS (physiological experiment) were less efficient in these children than in those in the control group.

## Experiment 1: emotion detection probe

The first study tested participants’ ability to recognize facial expressions with a high percentage of accuracy. (Note, establishing that children of this age group could recognize the expressions accurately was an important prerequisite for the valid assessment of ANS responses in experiment 2, in which we used the same set of stimuli. Thus, the use of facial expressions that were not easily recognizable by children of this age would render uninterpretable results obtained in the second experiment.)

### Materials and methods

#### Participants

The study involved 26 subjects. Eight children with MBS (MBS group, MBS 3 females, *M*_age_ = 9 years; SD = 2.3) were recruited at the Operative Unit of Maxillofacial Surgery, Head and Neck Department.

In Table 1, demographic data and clinical information concerning all participants with MBS are reported. The children’s medical history was confirmed with the treating physician prior to testing. The inclusion criteria for children with MBS were (1) a certified diagnosis of unilateral or bilateral facial paralysis [30, 31, 33] (we included unilateral paralysis based on previous studies demonstrating that patients with hemiparesis also show impairment in emotion recognition [65]) and (2) a score > 70 percentile on the Colored Progressive Matrices Test, CPM [64]. Exclusion criteria were (1) the presence of congenital limb malformations and (2) the presence of any psychiatric or physical illness at the time of participation.

**Table 1:** Demographic and clinical characteristics of participants with Moebius syndrome in experiment 1

Group	Age	Sex	Paralysis	Cranial nerves involved	Dysfunction	IQ
MBS01	11	Female	Bilateral	Abducens nerve (VI)	No lateral eye movements	100
				Facial nerve (VII)	Facial palsy	
				Hypoglossal nerve (XII)	Fasciculations or atrophy of the muscles of the tongue	
MBS02	11	Female	Unilateral left	Abducens nerve (VI)	No lateral eye movements	110
				Facial nerve (VII)	Facial palsy	
MBS03	11	Male	Bilateral	Facial nerve (VII)	Facial palsy	105
				Accessory nerve (XI)	Ipsilateral weakness in the trapezius muscle	
				Hypoglossal nerve (XII)	Fasciculations or atrophy of the muscles of the tongue	
MBS04	8	Male	Bilateral	Abducens nerve (VI)	No lateral eye movements	100
				Facial nerve (VII)	Facial palsy	
				Hypoglossal nerve (XII)	Fasciculations or atrophy of the muscles of the tongue	
MBS05	6	Male	Bilateral	Facial nerve (VII)	Facial palsy	100
MBS06	8	Male	Bilateral	Abducens nerve (VI)	No lateral eye movements	110
				Facial nerve (VII)	Facial palsy	
				Hypoglossal nerve (XII)	Fasciculations or atrophy of the muscles of the tongue	
MBS07	11	Male	Unilateral left	Abducens nerve (VI)	No lateral eye movements	120
				Facial nerve (VII)	Facial palsy	
MBS08	6	Female	Unilateral right	Abducens nerve (VI)	No lateral eye movements	100
				Facial nerve (VII)	Facial palsy	
				Hypoglossal nerve (XII)	Fasciculations or atrophy of the muscles of the tongue	

The control group consisted of 18 children (control group, CG 3 females, *M*_age_ = 9 years; SD = 1.4) who did not meet criteria for a clinical diagnosis of MBS, or present with any psychiatric or physical illness, or any other neurological disorder.

Participants’ legal guardians gave written informed consent for the experimental procedure, which was approved by the Ethics Committee of Parma (prot. 32074). Participation in the study was voluntary and the participants were not paid. The study was conducted in line with the Declaration of Helsinki 2013.

#### Stimuli

**Fig. 1:**
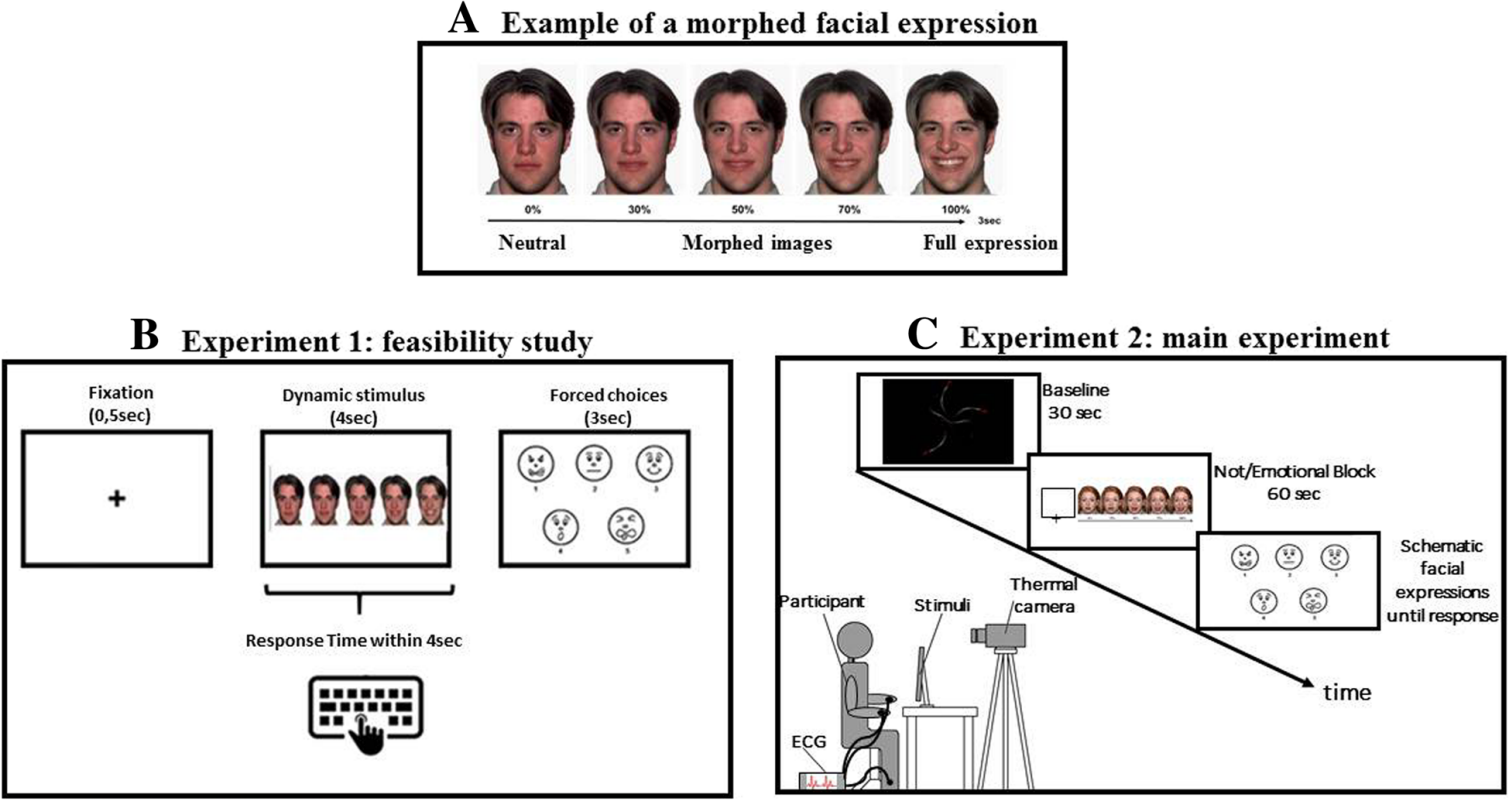
**a**An example of the morph steps. Each video clip showed an actor’s face morphing from the neutral expression to one of the five prototypical expressions (disgust, surprise, anger, happiness, and neutral). **b** Procedure of the experiment 1. Participants were presented with dynamic facial expressions one at a time. Each stimulus lasted for 4 s. When participants recognized the facial expression they pressed the space bar (stop button) and the stimulus disappeared (response time, RTs). RTs were considered an indicator of the time necessary to recognize the facial expression. Then participants were instructed to categorize each stimulus in a forced-choice procedure identifying the facial expression from a list of five stylized emotional faces (accuracy rate, RACC). **c** Procedure of the experiment 2. Participants were presented with 5 different blocks of morphed emotional faces (i.e., disgust, surprise, anger, happiness, and neutral). Each block lasted 60 s and was composed of different video clips representing the same emotion (12 facial expressions in total). Each video clip was preceded by 30-s baseline (i.e., a dynamic screensaver). At the end of each block participants underwent a control task (a forced-choice procedure identifying among five alternative pictures the emotion corresponding to the block previously seen)

Stimuli were short video clips lasting 4 s created using computer-morphing software (Abrosoft FantaMorph software package). Pictures (800 × 560 pixels) of four actors’ faces expressing five different emotions were selected from a set of validated pictures from the Nim Stim Face Stimulus Set [66]. Pictures consisted of four Caucasian actors’ faces (two males and two females) expressing four emotional facial expressions (i.e., disgust, surprise, anger, happiness) or a neutral facial expression (Fig. 1a).

Each video clip showed the transition from a neutral facial expression to an emotional one within the same actor (emotional video clips) or from a neutral face to another (neutral face, non-emotional video clips). In total, we created 60 stimuli (12 disgust, 12 surprise, 12 anger, 12 happiness, and 12 neutral stimuli). E-Prime 2.0 software (Psychology Software Tools, Inc.) was used for stimuli presentation.

#### Procedure

Once informed consent was obtained, participants were seated in a comfortable chair after being introduced to the experiment. Stimuli were presented centrally and the viewing distance was set at 60 cm from a 17 in. computer monitor (1024 × 768 at 75 Hz). Written instructions were presented on the screen before the beginning of each task and were read aloud to the participant by the experimenter.

Video clips were randomly presented one at a time. Each trial started with a fixation cross, presented for 0.5 s in the center of the screen. Each video clip lasted for 4 s (3 s of dynamic morph and 1 s of full emotion expression, Fig. 1b). Each stimulus was presented on a white background, with a dynamic morph starting from neutral and going to full facial expression.

Participants were told that the facial expressions appearing on the screen would look neutral at the beginning of the video clip and would gradually change to reveal one of five expressions (disgust, surprise, anger, happiness, and neutral expressions). They were asked to watch the facial displays change and to press the space bar to stop the video as soon as they thought they knew which expression the face was displaying. Participants were also instructed to maximize speed and recognition accuracy. When participants pressed the stop button, the stimulus disappeared and the response time was recorded as an index of the time necessary to recognize the facial expression. (The disappearance of the stimulus ensured that the response time reflected the actual recognition of the facial expression.) If participants did not press the space bar, the stimulus disappeared after 4 s.

After the stimulus disappeared, participants were instructed to categorize each stimulus in a forced-choice procedure identifying the facial expression from five options (stylized emotional faces). One practice trial was run, prior to 10 test trials (two trials for each emotion).

#### Statistical data analyses

We analyzed two dependent variables: response time (RTs) and accuracy rate (RACC). RTs were calculated as the time elapsed between the onset of the stimulus and the participants’ button press (recognition of a single facial expression). RACC rate was computed as the proportion of correct responses out of the total answers given (discrimination of facial expressions).

We excluded RTs less than 920 ms (less than 30% of morphing) in order to avoid anticipatory responses. RACC data were arcsine transformed prior to the analysis; values ranged from a minimum of zero to a perfect score of 1.57 (which is the arcsine of 1 [67]).

RTs were included as dependent variables into two mixed-design analysis of variance (ANOVA) in which “emotion” (five levels—disgust, neutral, surprise, anger, and happiness) was used as the within-subjects factor and “group” (two levels—MBS, CG) as the between-subjects factor. When the sphericity assumption was violated, Greenhouse–Geisser degrees of freedom corrections were applied. The probability value was set at *p* < 0.05 for all analyses. Partial eta squared (ηp^2^) was calculated as the effect size measure. Bonferroni post hoc tests were conducted following the two-way ANOVA.

Since many participants were 100% correct in recognizing some emotions, we considered only the total number of correct answers given by each group. The Kruskal–Wallis test was used as a non-parametric statistical procedure for comparing the RACC values of the two samples. The Statistical Package for the Social Sciences version 25 (SPSS, Chicago, IL, USA) was used to perform the analyses.

### Results

Table 2 contains means and standard deviations of participants’ RTs during emotional expression recognition for the MBS group (MBS) and control group (CG), respectively. Overall, disgust was the emotion that required the longest RTs (2349 ms), while happiness was the most rapidly recognized (1931 ms).

**Table 2:** Experiment 1: Mean and standard deviation (SD) of response times (in milliseconds) for neutral, disgust, surprise, anger, and happiness stimuli for the Moebius syndrome group (MBS) and control group (CG)

	Response time (ms)									
	Neutral		Disgust		Surprise		Anger		Happiness	
	Mean	SD	Mean	SD	Mean	SD	Mean	SD	Mean	SD
MBS	2282	437	2382	409	2208	492	2027	373	1920	232
CG	2089	413	2335	416	2170	339	2108	416	1936	396

Mixed ANOVA on RTs revealed a main effect of emotion (*F* (4, 96) = 9.9; *p* = 0.001; ηp^2^ = 0.29). Bonferroni post hoc *t* tests revealed that participants recognized happy video clips significantly faster (1931 ms) than disgust (2349 ms, *p* = 0.001), neutral (2149 ms, *p* = 0.014), and surprise (2182 ms, *p* = 0.003) video clips. Conversely, disgust was the emotion that took the longest RTs (disgust vs. neutral, *p* = 0.032; disgust vs. anger, *p* = 0.001). No significant group or interaction (group × emotion) effects were observed (*p* > 0.05).

Table 3 contains means and standard deviations of participants’ RACC rates for the recognition of emotional expressions. In general, the judgments of facial stimuli were highly accurate (mean RACC = 96% ± 4.7).

**Table 3:** Means and standard deviations (SD) of response accuracy rate for the recognition of each emotional expression in experiment 1 and at the end of each block in experiment 2 showed by Moebius Syndrome group (MBS) and control group (CG)

	Neutral		Disgust		Surprise		Anger		Happiness	
	Mean	SD	Mean	SD	Mean	SD	Mean	SD	Mean	SD
Experiment 1_Accuracy (%)										
MBS	96	8.9	79	24.3	96	6.4	96	9	95	14.7
CG	100	2	91	8.5	100	0	99	2.7	98	3.8
Experiment 2_Accuracy (%)										
MBS	69	0.5	85	0.4	100	0	100	0	92	0.3
CG	100	0	94	0.3	100	0	100	0	94	0.3

**Fig. 2:**
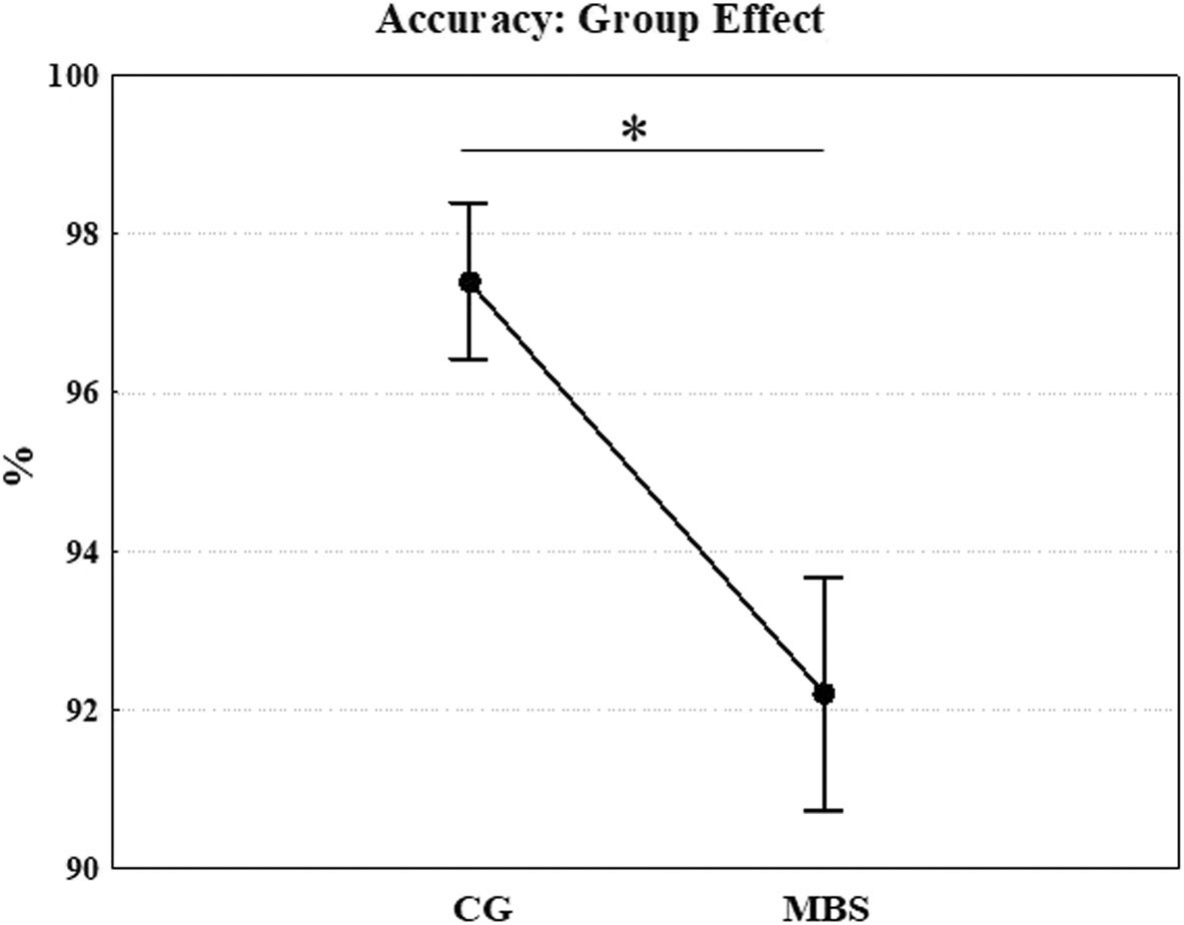
Experiment 1: Kruskal–Wallis test was used for comparing the RACC values of the two samples. Mean accuracy scores (%) for control (CG) and Moebius syndrome group (MBS) groups on recognition of five facial expressions are reported. *p < 0.05. Error bars represent SE (standard errors of the means)

The Kruskal–Wallis test comparing RACC values between the groups showed that RACC scores were significantly lower for MBS than CG (chi-square = 5.096; *p* = 0.024, MBS = 92.2%, CG = 97.4%, respectively; Fig. 2), indicating that, although they were very accurate, MBS participants’ performance in discriminating facial expressions was poorer than that of the control group.

### Discussion

Results of this first study revealed an absence of group difference in RTs, and this finding supports the hypothesis that children with MBS had a comparable level of emotion recognition to that of the control group. These results are in line with previous studies [50, 52] in which face stimuli were presented singly and exclude the possibility that children with MBS may have difficulty inspecting stimuli, because their response times were similar to those of the control group.

However, the RACC analysis showed that the control group was more accurate than children with MBS, suggesting some difficulties in the latter group in discriminating the facial expressions displayed. Although the high percentage of accuracy and the small number of participants preclude us from concluding that children with MBS have deficits in emotional recognition, the results nevertheless highlight some difficulty when required to identify facial expressions from an array of stimuli with emotional content (complex facial recognition task). We hypothesize that participants with MBS, not being able to benefit fully from motor simulation mechanisms, probably use alternative cognitive strategies, which may not be as effective as simulation (at least during early development) [29]. One of these cognitive mechanisms could involve strict, rule-based, strategies, in which memorized lists of characteristics defining emotional expressions are employed. Such strategies may lead children to look for the presence of these specific features when performing emotional perception tasks. For example, a “rule” for disgust could be “corners of the actor’s mouth turned down.” This characteristic, present both in disgust and anger, was efficient for recognizing the facial expression when present but was ineffective when choosing among multiple options when the stimulus was no longer present.

Our findings are also in line with the conclusion of Calder et al. [50] and Bate et al. [51] that, while difficulties in emotional face recognition are prevalent in individuals with MBS, they are not invariable. It is also possible that the higher number of errors we found in children with MBS might be related to the young age of participants. Thus, previous studies have focused on adults, while here we included children who have probably not yet fully developed other cognitive strategies for recognizing others’ emotions.

This study had a number of limitations. First, the small sample of patients and the limited number of facial expressions used limit the generalizability of our results. Second, the high level of accuracy in facial expression recognition observed in both groups may have masked group differences in recognizing specific emotions. In future, more refined measurements of emotion recognition and the use of more complex stimuli (e.g., morphed facial expressions of two or more emotions) could be useful in identifying subtler difficulties in children with Moebius syndrome. Further, these preliminary results will need to be further investigated using more complex experimental designs and a greater number of stimuli. Moreover, follow-up assessments should be carried out across development, from childhood to adolescence, in order to assess the improvement of facial expression recognition in these patients.

## Experiment 2: physiological experiment

In this study, we tested whether the responses of the ANS during emotional processing were altered in children with MBS compared to a control group. Specifically, we monitored the variations of facial temperature and the amplitude of RSA in children with MBS and controls when they were presented with 1-min-long videos depicting dynamically changing facial expressions, from a neutral face to one showing disgust, surprise, anger, happiness, or else remaining neutral. Given that children recognized the facial expressions used in experiment 1 with a high degree of accuracy, we used the same sets of stimuli in experiment 2 in order to measure the autonomic response to different emotional stimuli.

### Materials and methods

#### Participants

A new group of 13 children with MBS (MBS group, MBS 7 females, *M*_age_ = 8.7 years; SD = 2.8, see Table 4) participated in the study (see inclusion/exclusion criteria in experiment 1, the emotion detection probe study, participants section). The healthy control group (CG) consisted of 16 participants (6 females, *M*_age_ = 9.3 years; SD = 1.7). Participants’ legal guardians gave written informed consent for the experimental procedure, which was approved by the Ethics Committee of Parma (prot. 32074). Participation in the study was voluntary and the participants were not paid. The study was conducted in line with the Declaration of Helsinki 2013.

**Table 4:** Demographic and clinical characteristics of participants with Moebius syndrome in experiment 2

Group	Age	Sex	Paralysis	Cranial nerves involved	Dysfunction	IQ
MBS09	11	Female	Bilateral	Abducens nerve (VI)	No lateral eye movements	100
				Facial nerve (VII)	Facial palsy	
				Hypoglossal nerve (XII)	Fasciculations or atrophy of the muscles of the tongue	
MBS10	5.5	Female	Unilateral right	Abducens nerve (VI)	No lateral eye movements	80
				Facial nerve (VII)	Facial palsy	
MBS11	5.5	Female	Unilateral right	Abducens nerve (VI)	No lateral eye movements	100
				Facial nerve (VII)	Facial palsy	
				Hypoglossal nerve (XII)	Fasciculations or atrophy of the muscles of the tongue	
MBS12	10	Male	Bilateral	Abducens nerve (VI)	No lateral eye movements	80
				Facial nerve (VII)	Facial palsy	
				Hypoglossal nerve (XII)	Fasciculations or atrophy of the muscles of the tongue	
MBS13	9.5	Female	Bilateral	Abducens nerve (VI)	No lateral eye movements	110
				Facial nerve (VII)	Facial palsy	
				Hypoglossal nerve (XII)	Fasciculations or atrophy of the muscles of the tongue	
MBS14	13	Male	Unilateral left	Abducens nerve (VI)	No lateral eye movements	110
				Facial nerve (VII)	Facial palsy	
MBS15	6	Female	Unilateral left	Abducens nerve (VI)	No lateral eye movements	110
				Facial nerve (VII)	Facial palsy	
				Hypoglossal nerve (XII)	Fasciculations or atrophy of the muscles of the tongue	
MBS16	7	Male	Bilateral	Abducens nerve (VI)	No lateral eye movements	80
				Facial nerve (VII)	Facial palsy	
				Vestibulocochlear nerve (VIII)	Hearing loss	
				Hypoglossal nerve (XII)	Fasciculations or atrophy of the muscles of the tongue	
MBS17	8	Male	Bilateral	Abducens nerve (VI)	No lateral eye movements	100
				Facial nerve (VII)	Facial palsy	
				Hypoglossal nerve (XII)	Fasciculations or atrophy of the muscles of the tongue	
MBS18	8	Male	Bilateral	Abducens nerve (VI)	No lateral eye movements	110
				Facial nerve (VII)	Facial palsy	
				Hypoglossal nerve (XII)	Fasciculations or atrophy of the muscles of the tongue	
MBS19	12	Male	Unilateral left	Abducens nerve (VI)	No lateral eye movements	120
				Facial nerve (VII)	Facial palsy	
MBS20	5	Female	Bilateral	Abducens nerve (VI)	No lateral eye movements	100
				Facial nerve (VII)	Facial palsy	
				Hypoglossal nerve (XII)	Fasciculations or atrophy of the muscles of the tongue	
MBS21	12	Female	Unilateral left	Abducens nerve (VI)	No lateral eye movements	100
				Facial nerve (VII)	Facial palsy	

#### Stimuli

The sets of stimuli comprising different facial expressions used in this study were the same as those for experiment 1. Before measuring the impact of these stimuli on ANS reactivity, the recognition of each facial expression was carefully evaluated, as in the first study. We confirmed that the judgments of the facial stimuli were highly accurate (mean RACC = 96%) in both groups.

#### Procedure

Before the start of the experiment, each subject was left to acclimatize themselves for 10–20 min in a softly lit, sound-proofed, climate-controlled room (room temperature 23 ± 1 °C; relative humidity 50–55%; no direct sunlight or ventilation). Five different blocks of morphed emotional faces (i.e., disgust, surprise, anger, happiness, and neutral) were randomly presented to the subject (Fig. 1c). Subjects sat comfortably in a chair, without any restriction of their body movements.

In total, the participants observed 60 video clips divided into 5 experimental blocks. Each block was composed of different video clips representing the same emotion. Four video clips (two males, two females) each lasting 4 s (Fig. 1c) were repeated three times and shown in the same block (12 facial expressions in total). Each video clip was preceded by a fixation cross displayed in the center of the screen for 1 s. Thus, each block lasted for a period of 60 s and was randomly presented. A baseline (i.e., a dynamic screensaver) lasting 30 s preceded each block. In order to control participants’ attention, at the end of each block, an image with five-forced choice picture options appeared on the screen. It remained visible until the participant responded (Fig. 1c). The experimenter asked the subject to identify which of the five alternative pictures matched the emotion previously displayed in the block. Participants were instructed either to answer verbally or to point to the chosen image. The child’s answer was then noted on the experimental pre-prepared sheet.

During video clip presentations, the participant was asked to simply observe the stimuli. Participants’ fITI and ECG were recorded for the entire duration of the experiment. Thermal IR imaging was recorded by means of a digital thermal camera FLIR T450sc (IR resolution 320 × 240 pixels; spectral range 7.5–13.0 μm; thermal sensitivity/NETD < 30 mK at 30 °C). The acquisition frame rate was set to 5 Hz (5 frames/s). A remote-controlled webcam (Logitech webcam C170) was used to film children’s behavior to assure that they paid attention to the stimuli. The thermal camera was placed just above the screen used for the stimuli presentation, 1 m away from the participant’s face, and it was automatically calibrated and manually fixated to allow a frontal recording of the child’s face.

ECG was recorded using three Ag/AgCl pre-gelled electrodes (ADInstruments, UK) with a contact area of 10-mm diameter placed in an Einthoven’s triangle configuration (Powerlab and OctalBioAmp8/30, ADInstruments, UK). The answers given by the children at the end of each block were considered an index of accuracy and treated as in the emotion detection probe study (the “Statistical data analyses” section).

#### Thermal data analysis

Firstly, we performed a visual inspection of the changes in subjects’ thermal responses to provide a qualitative investigation of their autonomic responses throughout the experiment. Then, thermal variation, i.e., changes in cutaneous temperature was calculated for specific facial regions of interest (ROIs) [43, 68].

**Fig. 3:**
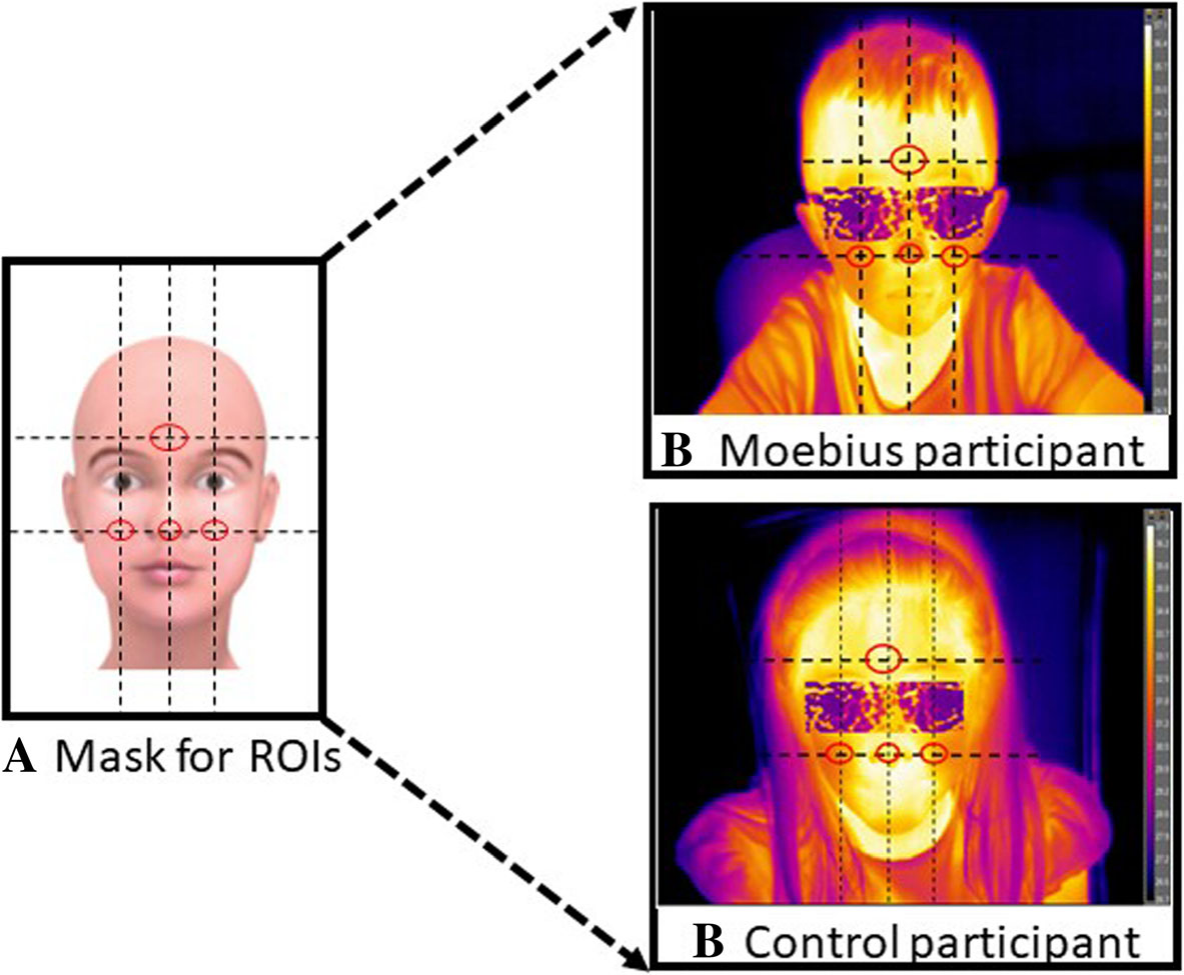
To extract information of affective nature, three regions of interest (ROIs) were used (tip of the nose, forehead, and cheeks). A mask was initially created (**a**) in which the ROIs were drawn to ensure that there was no variability across the size and shape of the ROIs among participants. Taking as a reference point the nose, we traced an imaginary straight line centered on the tip of the nose to locate the area of the forehead placed above the two eyebrows. Further horizontal lines passing through the center of the eyes and the tip of the nose allowed us to identify the area of the cheeks. The same mask was uploaded on each participant’s first frame and then a trained experimenter analyzed frame by frame the movements of the participant’s face during the experiment. Whenever the participant moved, the experimenter adjusted the position of each ROI on the participant’s face. If the participant’s movements did not allow the reposition of the ROIs, the frame was discarded. In **b**, we reported two example of thermal extraction (in the top panel a child with Moebius syndrome and in the lower panel a child belonging to the control group). In the images of participants, blurred masks (glasses) are included on the children’s face in order to occlude their identity

We performed a quantitative estimation of temperature variation in the following ROIs: nasal tip [44, 69, 70], cheeks [45], and forehead (Fig. 3 [43]). The shapes of ROIs did not vary in size across frames, and the temperature was extracted only when the face was at a direct angle to the camera [43, 71]. The same circular shapes were used for both groups. We initially created a mask in which the ROIs were drawn (Fig. 3a). We then took the tip of the nose as the reference point, this being an anatomical “landmark” that is easily identifiable in all subjects [68]. Subsequently, tracing an imaginary straight line centered on the tip of the nose, we located the area of the forehead positioned above the two eyebrows. Further horizontal lines that passed through the center of the eyes and the tip of the nose allowed us to identify the area of the cheeks.

Once the mask with the ROIs was created, it was uploaded for each participant’s first frame. Since the participants were free to move during the observation of the stimuli, one of the experimenters analyzed the movements of the participant’s face frame by frame during the experiment. If the participant moved, the experimenter adjusted the position of each ROI on the participant’s face, maintaining their relative positions constant. If the participant’s movements did not allow accurate repositioning of the ROIs, the frame was discarded. Thus, thermal signals were extracted and processed (Fig. 3b) by a trained coder through the use of tracking software, developed with homemade Matlab algorithms (The Mathworks Inc., Natick, MA) and validated in [72].

To avoid any possible noise or artifacts, thermal data were subsequently examined with PostTracking software. On average, we extracted 150 frames (30s) for each baseline pre-block, and 300 frames (60 s) for each experimental block (neutral, happiness, surprise, anger, disgust). Non-parametric Pearson correlations (Bonferroni adjusted) for the three ROIs yielded significant results (*α* = 0.05/3 = 0.02, Table 5) indicating that the three dependent variables were highly correlated.

**Table 5:** Experiment 2: Results of Pearson’s correlations based on the three ROIs

	Forehead	Cheeks	Nose
Forehead	1.0000	0.8762	0.6882
	*–*	*p* = 0.000*	*p* = 0.000*
Cheeks	0.8762	1,0000	0.5812
	*p* = 0.000*	*–*	*p* = 0.001*
Nose	0.6882	0.5812	1,0000
	*p* = 0.000*	*p* = 0.001*	*–*

*Pearson correlation was significant at the 0.05 level (two-tailed). Bonferroni corrected

To eliminate temperature changes that were unrelated to the experimental conditions and to reduce inter-subject variability, thermal values were obtained by subtracting the mean thermal values of each ROI during the pre-block baseline from the mean thermal values of the ROIs during each experimental block.

#### Statistical analyses

First, we checked that the one-way ANOVA performed on the neutral block thermal values (neutral facial expression) in the three ROIs did not show significant differences between groups (*p* > 0.05). Then, the temperature values for each emotional block (disgust, surprise, anger, and happiness) were subtracted from those for the neutral block [43]. Given that the temperature values of the three ROIs (forehead, cheeks, and nose) were significantly correlated (Table 5), we performed a multivariate analysis of variance (MANOVA) in which the dependent variables were the three face ROIs [73]. Thus, the effects of emotional stimuli observation on facial temperature were analyzed via a 4 × 2 MANOVA (emotion × group). The probability value was set at *p* < 0.05 for all analyses. Significant MANOVA findings are expressed using Wilks’ lambda (*Λ*) and effect size data (ηp^2^) were also provided for additional information. The Statistical Package for the Social Sciences version 25 (SPSS, Chicago, IL, USA) was used for all analyses.

#### RSA analysis

ECG data were converted and amplified with an eight-channel amplifier (PowerLab8/30; ADInstruments UK) and displayed, stored, and reduced with the LabChart 7.3.1 software package (ADInstruments, 2011). ECG was sampled at 1 kHz and online filtered with the Mains filter. Heart period was calculated as the interval in milliseconds between successive R-waves. The amplitude of RSA [expressed in ln (ms)^2^] was quantified with CMetX (available from http://apsychoserver.psych.arizona.edu), a software for calculating cardiac variability that produces data with a correlation near unity with those obtained using the method of Boher and Porges [74]. The amplitude of RSA was calculated as the variance of heart rate activity across the band of frequencies associated with spontaneous respiration (0.24–1.04 Hz for children below the age of 11 years and 0.12–0.40 Hz for children older than 11 years) [74]. ECG data for two subjects were discarded because of technical problems. The resting RSA value was the mean of each 30-s screensaver baseline that preceded each block (2.5 min in total). RSA reactivity refers to RSA values extracted from two epochs (each of 30 s) during 1 m of each experimental block and expressed as the difference from resting RSA.

#### Statistical analyses

To investigate the functional modulation between vagal regulation and external, social stimuli, we first performed a one-way ANOVA to test differences in the resting RSA between groups. A 5 × 2 repeated mixed ANOVA was performed on RSA reactivity with emotion (neutral, disgust, happiness, anger, and surprise) as a repeated measures factor and group (MBS vs. CG) as a between-participants factor. When the sphericity assumption was violated, Greenhouse–Geisser degrees of freedom corrections were applied. The probability value was set at *p* < 0.05 for all analyses. Partial eta squared (ηp^2^) was calculated as the measure of effect size. Bonferroni post hoc tests were conducted following the two-way ANOVA.

Pearson’s correlations were also calculated to assess RSA reactivity in relation to individual resting RSA in response to facial expressions and neutral stimuli [75]. Bonferroni corrections were applied (*α* = 0.05/5 = 0.01). The Statistical Package for the Social Sciences version 25 (SPSS, Chicago, IL, USA) was used to perform all the analyses.

### Results

The Kruskal–Wallis test on RACC (the answers given by the children at the end of each block and considered an index of accuracy) between groups showed that scores were nevertheless significantly lower for MBS than CG (chi-square = 4.107; *p* = 0.043, MBS = 92.2%, CG = 97.4%).

**Fig. 4:**
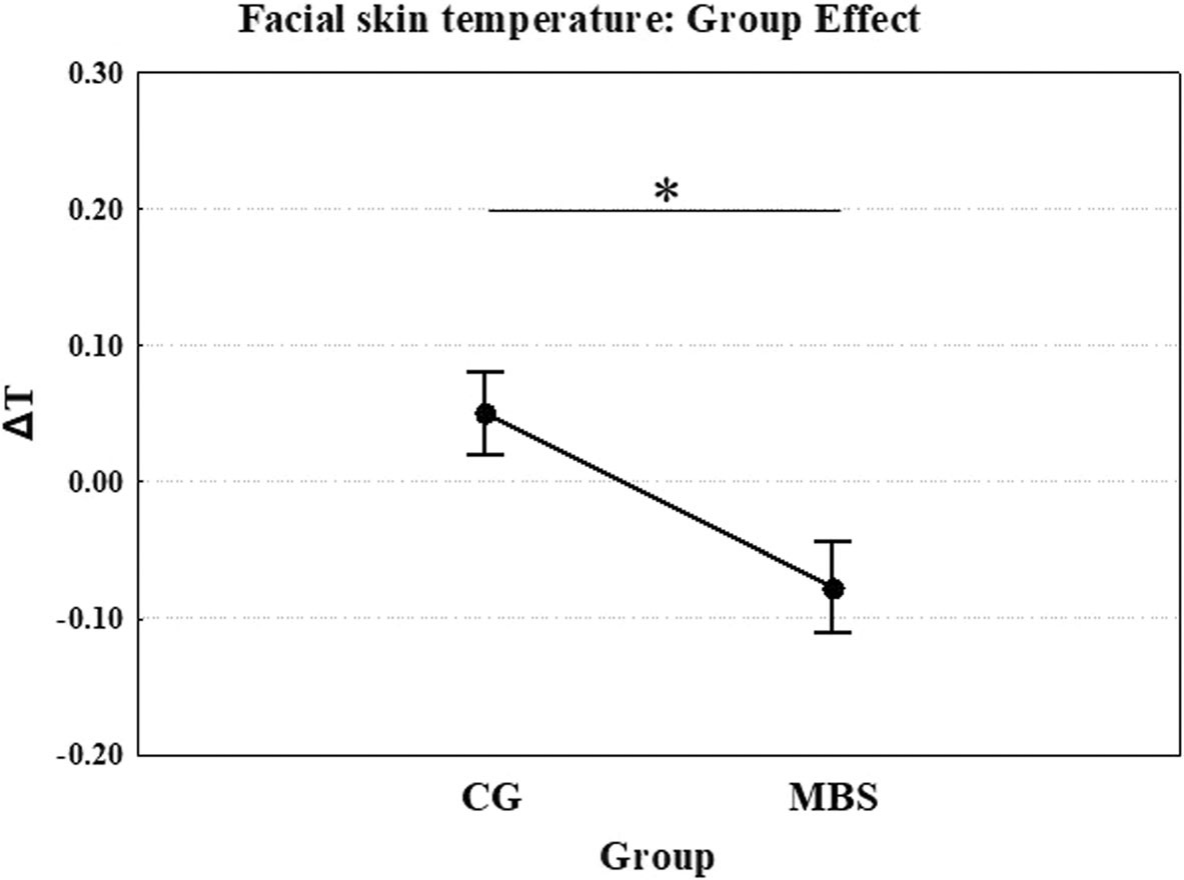
Experiment 2: Mean temperature as a function of group (children with MBS and control group, CG) in all ROIs. *p < 0.05. Error bars represent SE (standard errors of the means)

Consistent with the study hypothesis, thermal analysis showed a significant multivariate main effect of group (*Λ* = 0.915, *F* (3, 106) =3.27; *p* = 0.024, ηp^2^ = 0.085). Specifically, children with MBS (MBS − 0.077 ΔT) showed significantly lower thermal variation than the control group (CG 0.051 ΔT) while watching emotional stimuli (Fig. 4). No overall significant multivariate main effects of emotion (*p* = 0.635) or interaction with group (*p* = 0.907) were observed.

**Fig. 5:**
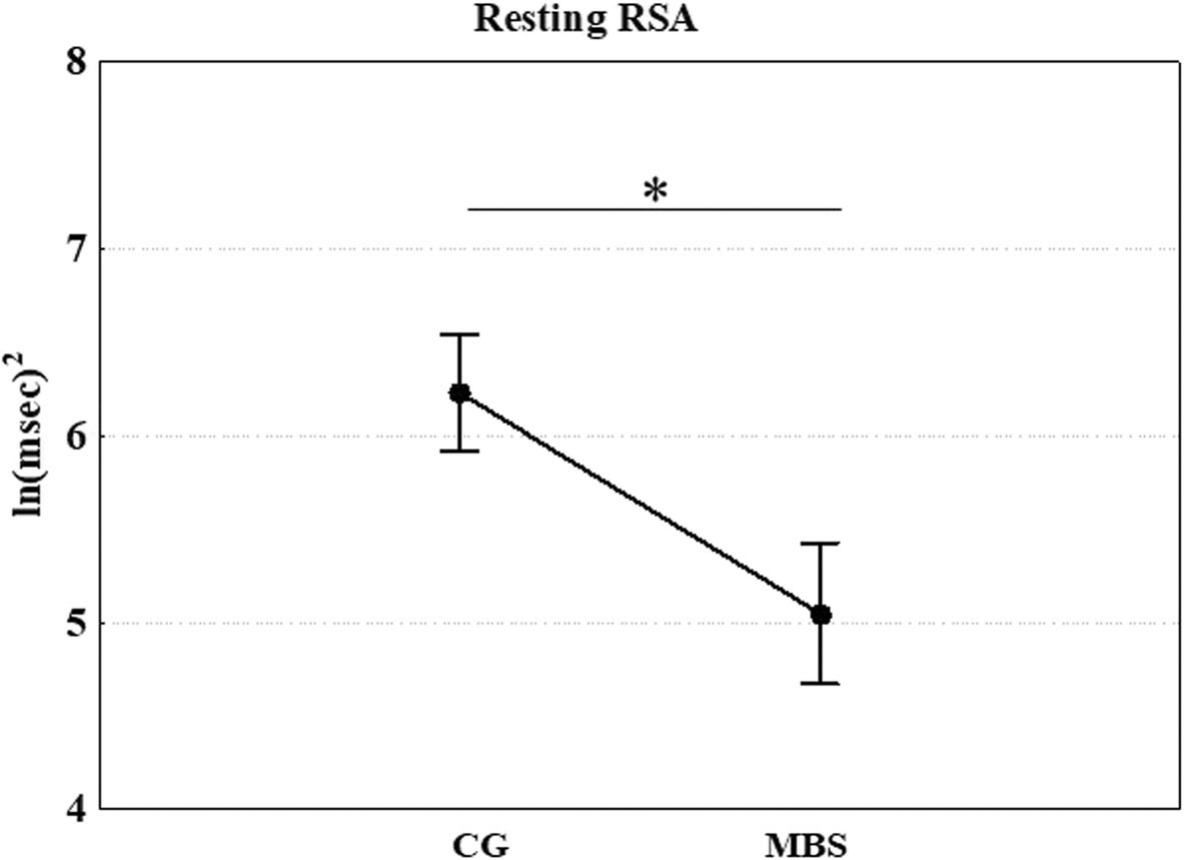
Experiment 2: Mean values of resting RSA as a function of group (children with MBS and control group, CG). *p < 0.05. Error bars represent SE (standard errors of the means)

Similar results were found when comparing the groups in terms of resting RSA. Thus, resting RSA was significantly higher in CG in comparison to MBS (*F* (1, 25) = 5.805; *p* = 0.024; ηp^2^ = 0.188, Fig. 5). The repeated mixed ANOVA performed on RSA reactivity did not show significant emotion or group main effects (*p* = 0.526 and *p* = 0.614, respectively), and there was no significant (group × emotion) interaction (*p* = 0.454).

**Fig. 6:**
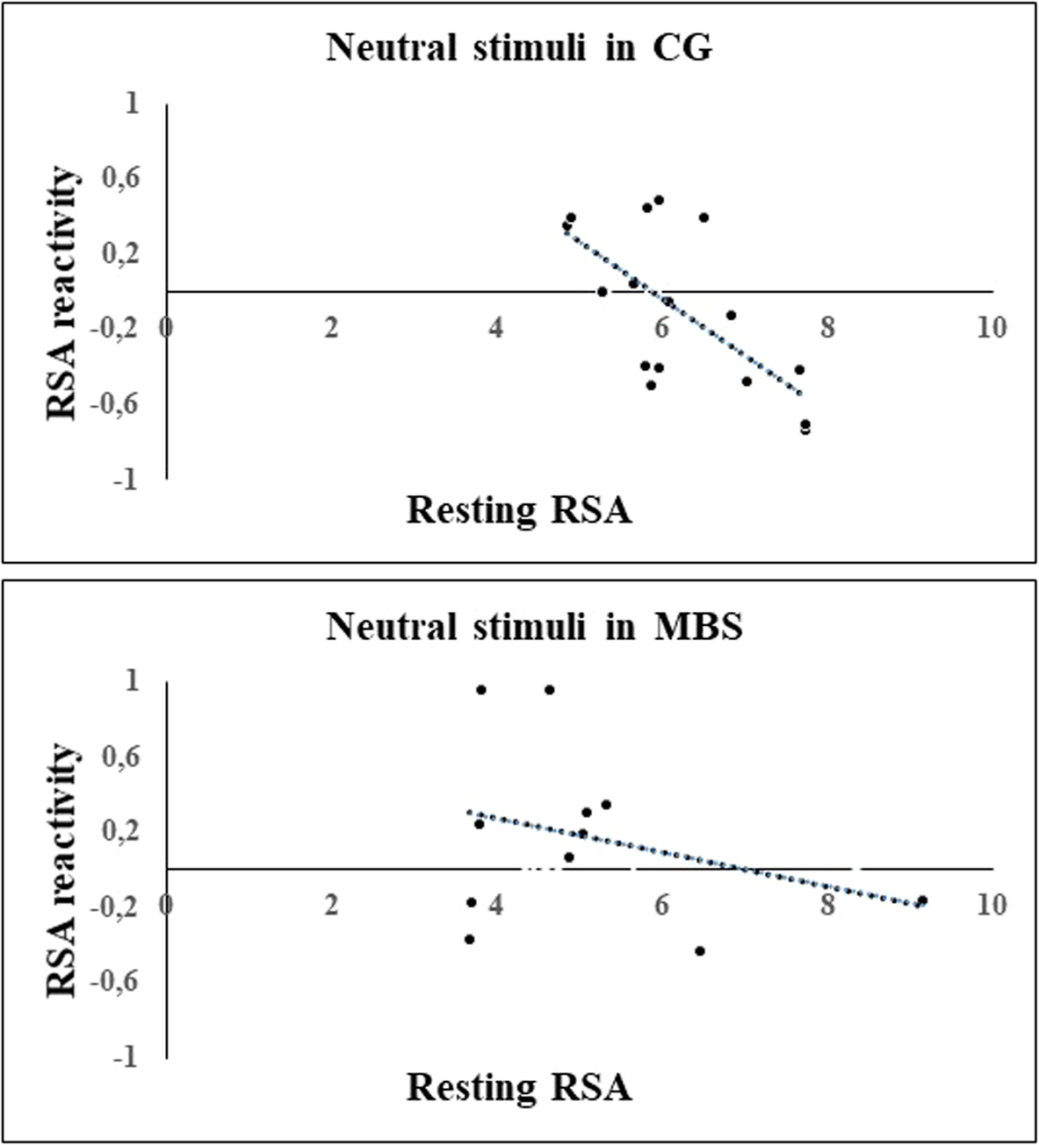
Experiment 2: Plots of correlations between baseline and RSA reactivity values recorded during the observation of neutral stimuli for children with Moebius syndrome (MBS) and control group (CG) (Bonferroni corrected *p* < 0.01)

In order to determine whether there was a significant association between resting RSA and RSA reactivity, we performed two correlational analyses (one for each group) between the resting RSA and the RSA reactivity values for each condition. Pearson’s correlations demonstrated a significant negative correlation between baseline RSA and RSA reactivity in CG only, in response to the neutral condition (*r* =  − 0.665, Bonferroni corrected *p* = 0.005, Fig. 6). No other significant correlations for either MBS or CG were found.

### Discussion

In this second study, we focused on the contribution of the capacity for facial mimicry to autonomic regulation in response to others’ emotions. We addressed this issue by studying a population of children with MBS, a rare neurological disorder that primarily affects the muscles controlling facial expressions. Individuals with MBS are born with facial muscle paralysis and an inability to produce facial expressions. This makes them the ideal population to study whether autonomic responses to emotional stimuli are affected by the inability to simulate the emotions of others from birth.

We recorded facial thermal changes and ECG during the observation of dynamic facial expressions. We found a significant difference in facial thermal responses between groups. More specifically, consistent with previous studies, the control group showed greater thermal responses to emotional than to neutral stimuli relative to the Moebius group. By contrast, with respect to the neutral condition, Moebius children showed a skin temperature decrease, a response that is commonly associated with sympathetic activation in response to stressful, painful, or frustrating situations [46, 72]. This lower autonomic response of the MBS group did not vary between the different ROIs.

Contrary to what we expected, thermal responses did not turn out to differ between the various facial expressions displayed. Our findings are, therefore, at variance with previous demonstrations of the capability of thermal IR imaging to capture physiological thermal variations in relation to different emotional states. Thus, in the study by Merla and Romani [46], participants were exposed to a stressful task, and the largest temperature variations were reported for subjects who were more influenced by the judgment of others. Similarly, interpersonal social contact and sexual arousal have been shown to result in an increase in facial temperature [46, 76]. Temperature variations have also been found during stressful, fearful, painful, and guilty experimental situations [44, 46, 69]. Together, these studies show that exposure to a wide range of stimuli and situations results in large variations in autonomic system reactivity.

In contrast to previous studies, the stimuli used in our study were presented for a relatively short time period, and while they did, overall, elicit some arousal responses, these were minimal in magnitude and showed no specificity relative to the type of emotion. It is possible that a habituation effect due to repeated visual presentation within the same context could have leveled out potential thermal differences between emotional stimuli in our study. Future studies should explore in more depth the thermal responses of children with MBS in response to different types of emotional stimuli in order to understand whether this methodological approach is sensitive enough to detect autonomic differences between different emotions.

With regard to other indices of physiological regulation-resting RSA and RSA reactivity, our results demonstrated a significant group difference in the former that might reflect less predisposition in children with MBS to react to social stimuli and, in general, to environmental changes. Indeed, higher resting RSA indicates greater parasympathetic activation that promotes social interaction [37]. Interestingly, children who exhibited high resting RSA have been shown to exhibit greater empathic concern or helping [77]. By contrast, low resting RSA is considered a risk factor for anxiety, depression [78], trait hostility, and autism [79] and, more generally, can be considered a physiological response to environments that are perceived to be threatening. We also found a significant relation between resting RSA and RSA reactivity during the observation of neutral stimuli in the control group, whereas MBS group children seemed not to modulate their autonomic responses during this condition with respect to baseline level. In other words, in control participants only, the higher the RSA value at baseline, the stronger the RSA reactivity (i.e., RSA suppression) during the visualization of neutral facial expressions, a result that suggests these children recognized the neutral facial expressions as non-emotional stimuli and consequently modulated their ANS responses accordingly.

Findings from this second experiment also indicate that, compared to the control group, MBS is associated with both lower resting RSA and more dysfunctional RSA reactivity across conditions. It is interesting to consider that deficits in emotion regulation are common to other psychiatric conditions [62, 63], especially autism. Specifically, children with autism spectrum disorders have been shown to be slower in emotion recognition [80] and to have lower amplitude RSA [81]. These findings emphasize the role of ANS indexes in emotion regulation capabilities and suggest that abnormal ANS responses could be the basis of reduced social skills in these children [56, 66]. While further data are clearly necessary in order to investigate such a possible link, it is nevertheless interesting to note that some studies indicate that these children show deficits in social interaction and self-regulation in social contexts [44, 67].

The results of this study are consistent with simulation and embodiment theories of emotions [8, 9]. Thus, the simulation of other people’s facial configuration is held to trigger matched motor programs and their associated affective states, allowing emotion recognition [83, 84]. Accordingly, when the facial feedback is not available (as in the case of MBS), the response of the ASN is reduced [29, 85]. We suggest that, without the benefit of the capacity for facial mimicry, the identification of changes in an emotional face could instead arise from a stored representation of the visual perception of the dynamic movements of the face and the memorized characteristics of the corresponding emotion, which have been learned through associative processes (i.e., in the case of a happy face, the general configuration of smiles can be identified around the corner of the lips with exposure of the teeth). This could lead children with MBS to search, at cognitive level, for those specific characteristics that somehow affect the autonomic responses associated with the processing of others’ emotion. Thus, in addition to supporting the activation of shared facial motor programs, facial mimicry may contribute to the processing of visceromotor responses typically associated with the recognition of emotion [29].

Finally, in experiment 2, we observed a significant group difference in the responses at the end of each emotional block. Although such assessment was part of a control task, children with MBS nevertheless showed some difficulty in labeling the emotion just as observed. These results suggest some interesting possibilities, especially in relation to the results that emerged from the first study. Thus, in experiment 1, we showed that children with MBS were able to recognize facial expressions presented one at a time as fast as the control group. They were also accurate in labeling each facial expression (92%), despite the fact that the level of their performance was lower than that of the control group (97%). Consequently, in experiment 2, we expected that children with MBS would not show any difficulty in reporting what emotion they had seen, especially in view of the fact that stimuli representing the same emotion were presented several times in the course of the task (1 min of the same facial expression was presented in video sequences, each lasting 4 s). Instead, children with MBS showed lower levels of accuracy than controls. This highlights possible difficulties on the part of these patients in retaining information relating to the emotional aspects of facial configurations observed in the video. Interestingly, a recent study [86] showed that in healthy subjects, in whom facial mimicry was experimentally blocked, there was an impairment of the visual working memory mechanism for facial expressions. Although our results support the hypothesis of a link between facial mimicry, ANS activity and the facial recognition process, we cannot yet specify whether the link is mediated by sensorimotor mechanisms involved in the simulation process, which are somehow impaired in MBS children; by a purely visual memory system; or by an interaction between the two.

## Conclusion

Our results suggest that children with MBS have a less responsive parasympathetic system during observation of social stimuli compared to the control group. We suggest that the lack of motor simulation caused by peripheral facial paralysis had an impact on the ANS reactivity of these children, implying an altered capacity for processing emotional stimuli.

The link between motor simulation and ANS reactivity is supported by previous neuroimaging studies. These have demonstrated how both the production and the observation of an emotional facial expression activate not only specific motor and premotor cortical regions, but also brain areas directly involved in both visceromotor responses and the processing of the emotional valence of stimuli, such as the anterior insula, the anterior cingulate cortex, and the amygdala [24, 25, 28]. The recruitment of both cortical motor and subcortical structures while observing others’ social behavior [12, 25, 87] is thought to implement a mapping of the visual representation of an action or gesture to its corresponding motor representation [15, 88, 89]. Such sensorimotor mapping likely plays a fundamental role in the recognition of others’ behaviors and emotions, at a somatomotor level, as well as at the level of bodily changes (e.g., piloerection, heart rate changes, vasoconstriction) which are typically associated with emotional responses during first-person experiences. The capacity to share the inner aspects of emotions is the key to activating empathic responses and, in general, it is a necessary mechanism in the everyday regulation of social interactions [8, 9, 16, 21, 90, 91]. Consequently, the absence of the capacity for facial mimicry (as in the case of individuals with MBS) may impair not only facial expression recognition, but also related autonomic and somatic responses [8, 29, 85].

Our findings have important implications for our understanding of the emergence and development of emotional communication in infants and children. Considering that MBS is a congenital neurological condition present from birth, it is likely that the mild deficits both in emotion recognition and in ANS responses to emotion observation could also affect early social interactions between the infant and their caregivers. Thus, many studies have demonstrated the importance of the quality of the parent-child relationship in children’s emotion regulation capabilities [92, 93] and how, after birth, infant social expressiveness is accompanied by a highly organized, specific set of parental behaviors. Parents respond highly selectively to infant social cues by mirroring them and positively marking their occurrence with salient signals (e.g., smiles, eyebrow flashes) [94]. It has been also shown that such early interactions are critical for emotional attunement and self-regulation, as well as for the increase in social expressions in later development [94–97]. Other studies show that when infant social signals are perturbed by anatomical anomalies, such as cleft-lip, mothers tend to diminish their mirroring responses to infant social expressions, thereby impacting the development of infant social expressiveness [98]. Thus, the biological condition of impaired facial motor activity and its impact on early social interactions might both contribute to the social deficits of Moebius patients described in several studies [82, 99].

Because of the rarity of the syndrome, we could only include a small number of participants, and this precludes generalization of our results. For future studies, the research question should be addressed in a larger sample. Nevertheless, these data highlight the importance of studying the autonomic responses of children with MBS in different social contexts, where their decreased autonomic activation in response to the observation of others’ facial expressions could, at least in part, account for some of the difficulties of these children during social interactions.

## Data Availability

The dataset used and/or analyzed during the current study are available from the corresponding author upon reasonable request.

## Abbreviations

ANS: Autonomic nervous system
CG: Control group
ECG: Electrocardiography
fITI: Functional infrared thermal imaging
MBS: MBS group
MBS: Moebius syndrome
MNS: Mirror neuron system
RACC: Accuracy rate
ROIs: Region of interest
RSA: Respiratory sinus arrhythmia
RTs: Response time
